# Supplementary material for: Observation of gapped state in rare-earth monopnictide HoSb
Source: Sci Rep. 2020 Jul 31;10:12961. doi: 10.1038/s41598-020-69414-z (PMC7395779; doi:10.1038/s41598-020-69414-z)
Supplement: Supplementary file 1 — Supplementary information. [file 41598_2020_69414_MOESM1_ESM.pdf]

Supplementary Information for
Observation of gapped state in rare-earth monopnictide HoSb

M. Mofazzel Hosen,[1] Gyanendra Dhakal,[1] Baokai Wang,[2] Narayan Poudel,[3] Bahadur Singh,[2] Klauss Dimitri,[1] Firoza Kabir,[1] Christopher Sims,[1] Sabin Regmi,[1] William Neff,[1] Anan Bari Sarkar,[4] Amit Agarwal,[4] Daniel Murray,[3] Franziska Weickert,[5] Krzysztof Gofryk,[3] Orest Pavlosiuk,[6] Piotr Wiśniewski,[6] Dariusz Kaczorowski,[6] Arun Bansil,[2] and Madhab Neupane*[1]

[1]*Department of Physics, University of Central Florida, Orlando, Florida 32816, USA*

[2]*Department of Physics, Northeastern University, Boston, Massachusetts 02115, USA*

[3]*Idaho National Laboratory, Idaho Falls, Idaho 83415, USA*

[4]*Department of Physics, Indian Institute of Technology, Kanpur 208016, India*

[5]*National High Magnetic Field Laboratory, Los Alamos, New Mexico, 87545, USA*

[6]*Institute of Low Temperature and Structure Research, Polish Academy of Sciences, 50-950 Wrocław, Poland*

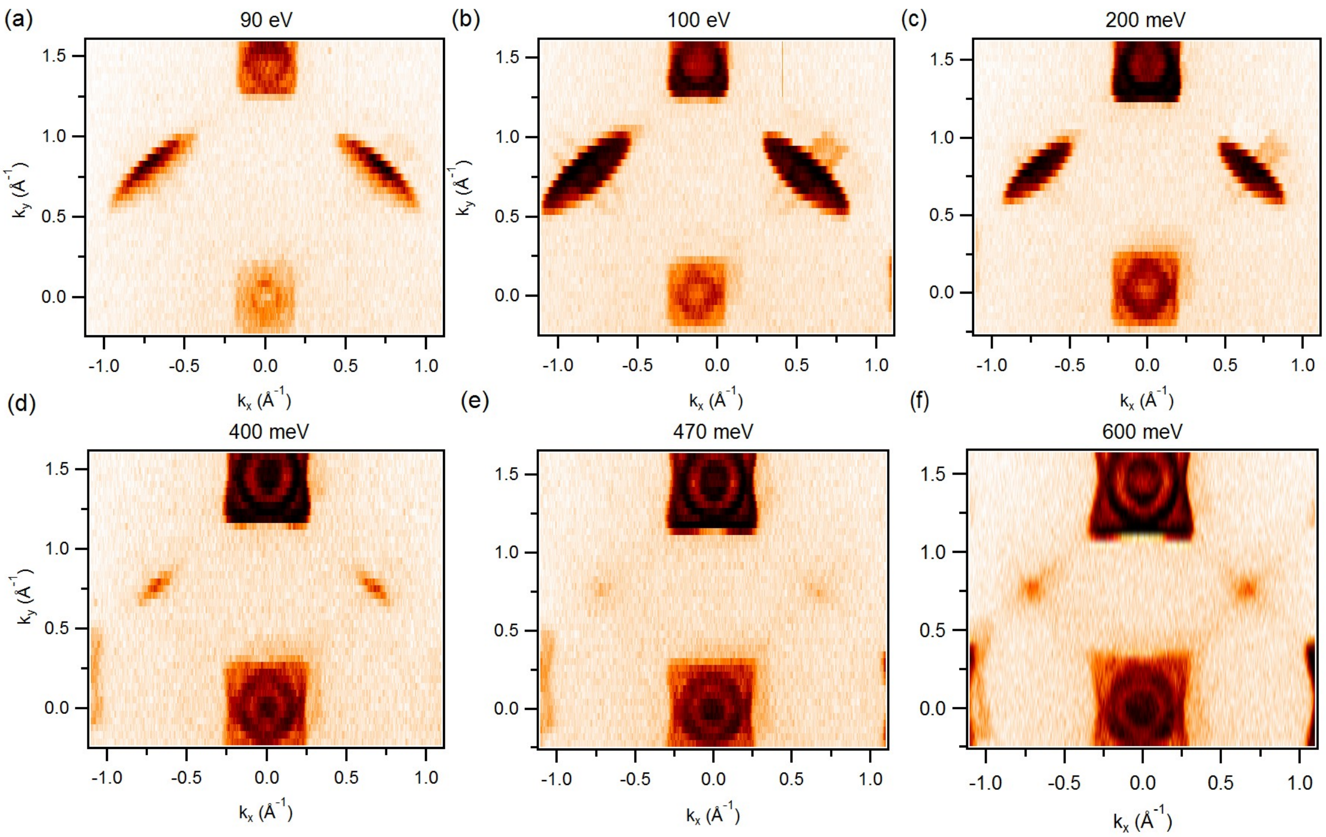

Supplementary figure 1: **Fermi surface and constant-energy contour plots.** (a),(b) Fermi surface maps of HoSb using various photon energies (noted in the plots) on a different batch of samples than those discussed in the main text. Photon energies are noted in the plots. (c)-(f) Constant energy contour plots at various binding energies for 100 eV photon energy. Experiments were performed at the ALS beamline 4.0.3 at a temperature of 18 K.

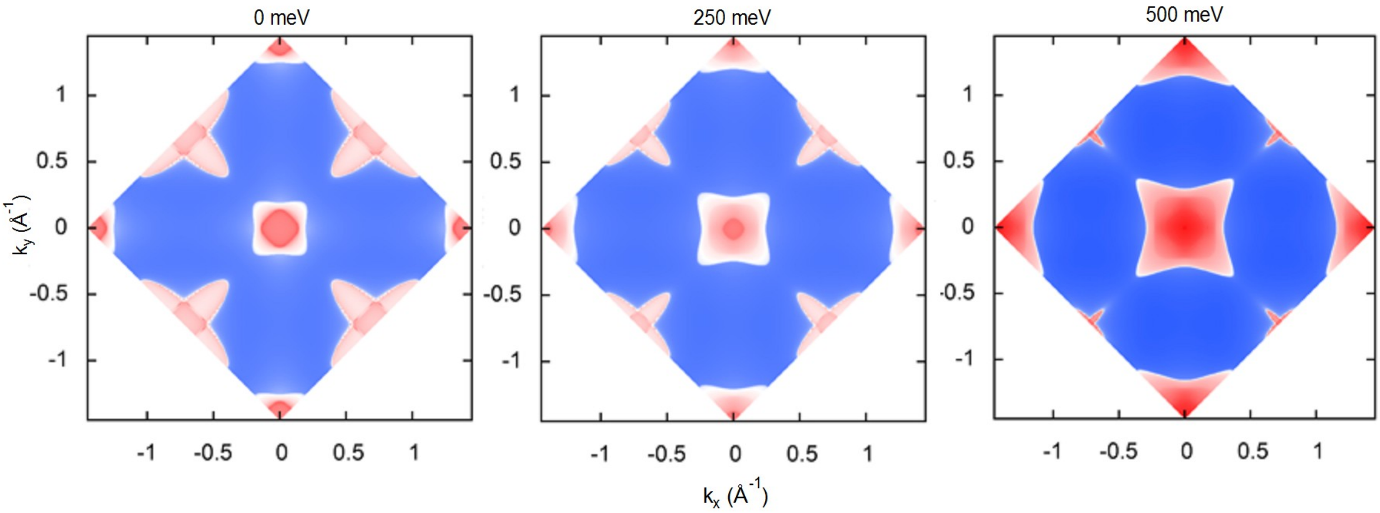

Supplementary figure 2: **Calculated Fermi surface and constant energy contour plots of HoSb.** Calculated Fermi surface and constant energy contour plots comparison of HoSb for various values of the binding energy (marked on the plots).

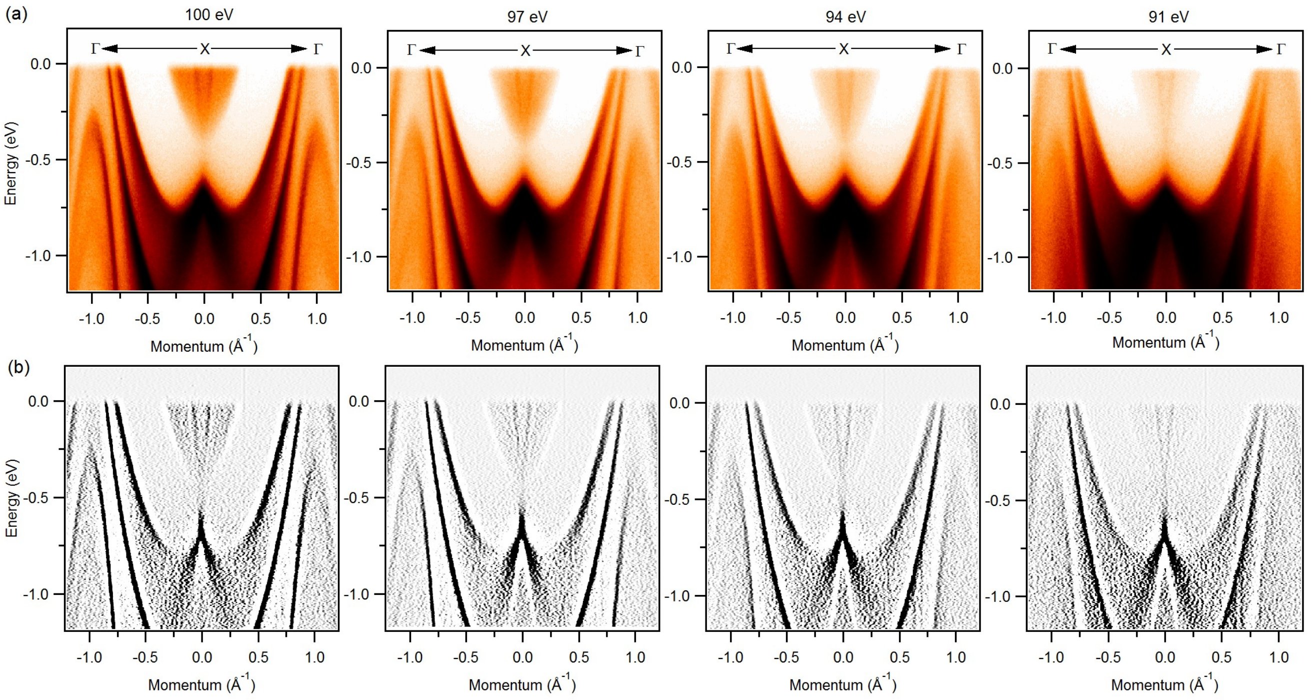

Supplementary figure 3: Dispersion maps along the Γ-M-Γ high symmetry direction. (a),(b) Measured dispersion maps along the Γ-X-Γ direction at various photon energies. (b) Second derivative plots for the spectra in (a) obtained by using the curvature methods . Photon energy values are noted in the plots. Experiments were performed at the ALS end-station 4.0.3 at a temperature of 18 K.

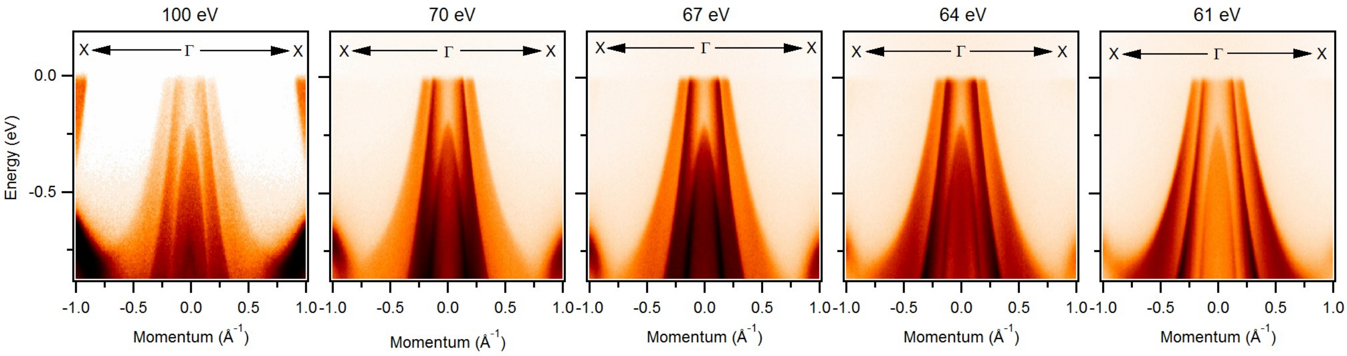

Supplementary figure 4: **Observation of bulk bands around the zone center.** Photon energy dependent dispersion maps along the X-Γ-X direction. Bands around the zone center (Γ) show notable dispersion as a function of incident photon energy. Measurements were performed at the ALS beamline 4.0.3 at a temperature of 18 K.

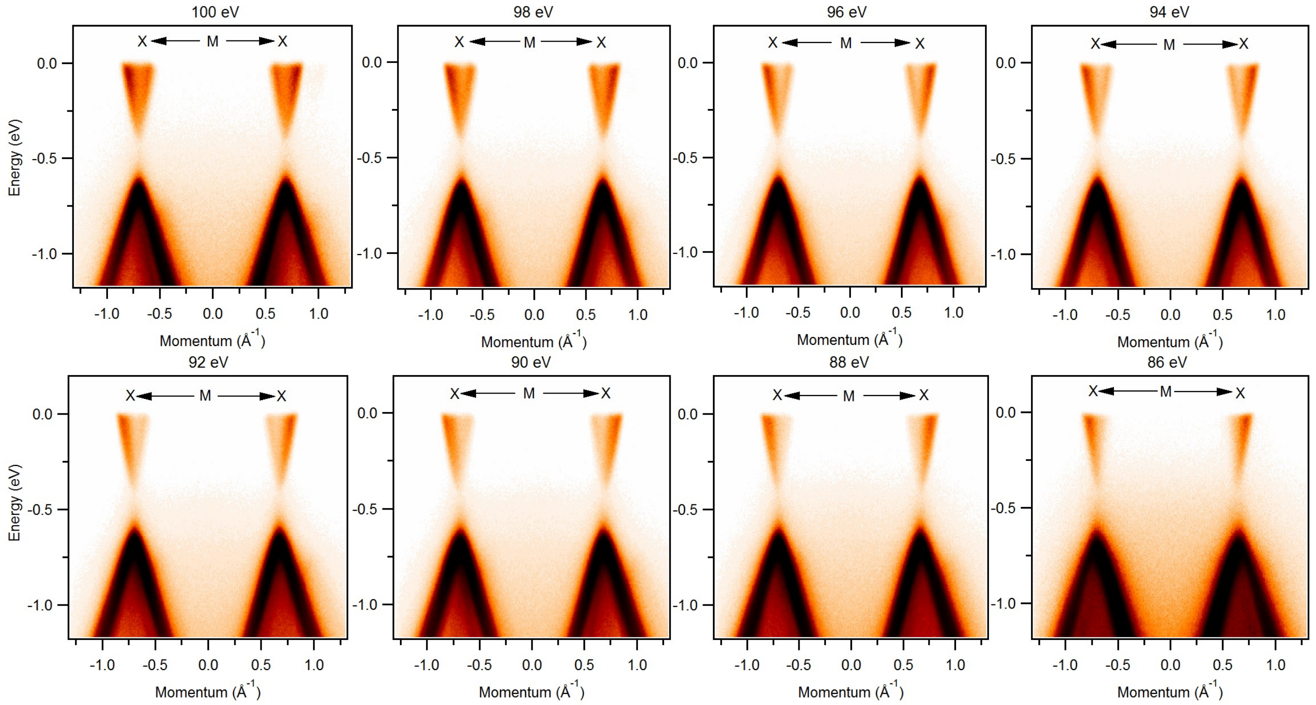

Supplementary figure 5: Experimental photon energy dependent dispersion maps along the X-M-X direction. Measured dispersion maps along the X-M-X direction using various photon energy with 2 eV energy steps from 100 eV to 86 eV as noted over the plots. Measurements were performed at the ALS beamline 4.0.3 at a temperature of 18 K.

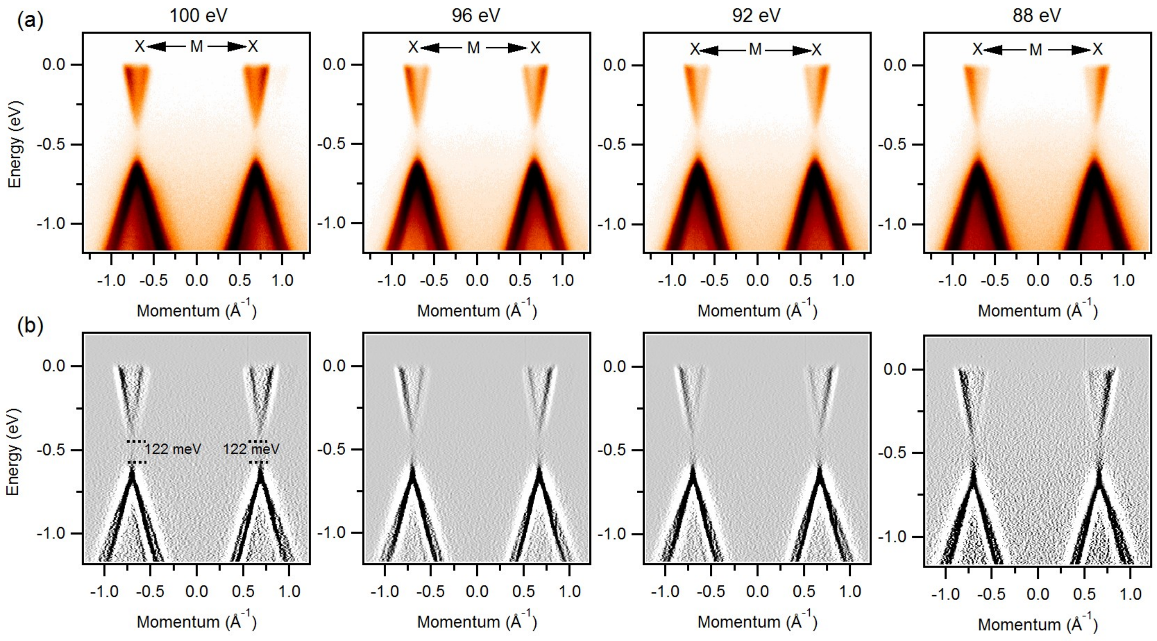

Supplementary figure 6: **Confirmation of gapped state**. (a),(b) ARPES measured dispersion maps along the high symmetry X-M-X direction and second derivative plots with different photon energies, respectively. Gapped state can be clearly seen. All measurements were performed at the ALS beamline 4.0.3 at a temperature of 18 K.

Supplementary Note 1

Fermi surface and constant energy contour plots of HoSb

We measured a second batch of samples to access the robustness of our Fermi surface and electronic structures results. Supplementary Figure (SF) 1 shows the observed Fermi surface (SF. 1(a)-(b)) and the related constant energy contour plots (SF. 1(c)-(f)) for the new measurements. Like the Fig. 1 in the main text, Fermi surface consists of an outer diamond and an inner circular pocket at the Γ point (zone center) and two concentric elliptical pockets at the X point of the Brillouin zone (BZ) (SF. 1(a)-(b)). Importantly, at a higher binding energy (∼470 meV), we observe that the elliptical-pocket evolves into a point-like feature. Supplementary Figure 2 shows the corresponding calculated Fermi surface and the related constant energy contour plots. An excellent agreement is seen between the experimental data and theoretical predictions.

Supplementary Note 2

Observation of the gapped state

In order to determine the origin of the bands near the zone center and the corner of the BZ, we performed photon energy dependent dispersion maps around these high-symmetry points. Supplementary Figures 3(a) and,(b) show the measured photon energy dependent dispersion maps and their second derivative plots along the Γ-X-Γ direction, respectively. Around the Γ point, two hole-like bands cross the Fermi level while at the X point, we observe a nearly linearly dispersive feature. To confirm the origin of the bands near the Γ point, we present more photon energy dependent dispersion maps in Supplementary Fig. 4 over a wide energy window. Here, one can clearly observe the photon energy dependent dispersive nature of the bands, so that the hole-like bands at around the zone center are bulk originated. Furthermore, from Supplementary Figure 3(b), we see a clear gap at the X point. In order to further confirm the gapped state at the X point, we present photon energy dependent dispersion maps along the X-M-X direction in the supplementary Fig. 5. The gapped state is seen consistently at all photon energies. In order to observe the gap size more vividly, we present measured dispersion maps and their second derivative plots in Supplementary Fig. 6. From panel 6(b), we approximate the band gap size around 120 meV. Moreover, the bulk state is once again clearly observed to be gapped which is consistent with the main text results.
